# Current Advances in Pediatric Sleep Medicine, Vol. 1

**DOI:** 10.3390/children13091175

**Published:** 2026-09-01

**Authors:** Ekkehart Paditz, Silke Anna Theresa Weber

**Affiliations:** 1 Center for Applied Prevention, 01307 Dresden, Germany; 2 Faculdade de Medicina (Câmpus de Botucatu), Departamento de Especialidades Cirúrgicas e Anestesi-Ologia, Universidade de São Paulo, Sao Paulo 05508-900, Brazil; silke@fmb.unesp.br

Although, at present, there is no holistic and transdisciplinary understanding of ‘sleep in infancy, childhood and adolescence’ [1,2,3,4,5,6], the contributions collected in this Special Issue, including nine articles by authors from Belgium, France, Spain, Austria, Germany, India and Australia, have helped to advance us towards achieving this goal. Readers may be surprised by the diversity of current perspectives and findings presented in these articles, which have already been viewed 45,296 times and have received as many as 21 citations in renowned specialist journals.

The authors included in this volume present compelling findings related to several areas of pediatric sleep medicine. Their contributions are as follows:

- Contribution 1: The causes and consequences of the only seemingly ‘physiological’ postnatal melatonin deficiency have been investigated for the first time in a systematic review. According to the review, the pineal gland is present at birth but not yet functional, as its innervation and the intracellular structures responsible for the circadian synthesis and secretion of melatonin only develop postnatally during the first few months of life. The clinical consequences of this finding, along with potential solutions, are presented by the author. This paper has already been cited 23 times and viewed 12,499 times within a short period of time.
- Contribution 2: In their systematic review, Romero-Peralta et al., from Spain, are the first to highlight the significant differences between girls and boys with obstructive sleep apnea (OSA).
- Contribution 3: Müller-Hagedorn et al., from France and Germany, present a comprehensive narrative review on the contribution of orthodontics to the widening of the upper airways in children and adolescents with OSA and anatomically related airway narrowing, such as micrognathia or midface hypoplasia.
- Contribution 4: Drawing on the findings of a narrative review and their own extensive clinical experience from both ENT and pediatric perspectives, Stuck and Schneider, from Germany, advocate for a ‘calculated wait-and-see’ approach for children and adolescents with OSA with regard to surgical treatment via adenoidectomy and/or ‘partial tonsillectomy’, provided this appears justifiable given the severity of OSA.
- Contribution 5: From a neonatological perspective, the sensory needs of preterm infants in intensive care units should be taken into account to a far greater extent than is currently the case. In this fifth contribution, the development of stable sleep–wake rhythms is promoted and supported as far as possible in the interests of optimal maturation of the brain’s cognitive and neural functioning.
- Contribution 6: In their retrospective analysis, Schlarb et al. demonstrate that, in 15–24 percent of the 499 cases studied, insomnia in children and adolescents was associated with nightmares.
- Contribution 7: The seventh contribution presents an interesting study protocol from Belgium for assessing the effectiveness of orofacial myofunctional therapy in children with Down’s syndrome and OSA.
- Contribution 8: In their article, Upreti and Frass, from India and Austria, present the first systematic review of the efficacy of homeopathic treatments in children and adolescents with defined sleep disorders.
- Contribution 9: In the final contribution, the significance of drug-induced sleep endoscopy (DISE) has been analyzed in 19 children with OSA and multiple rare syndromes with residual OSA, providing valuable insights for surgical planning.

Already having received 45,296 views and 43 citations, these studies have attracted significant international interest (Table 1). Fifty-six tables, figures and graphical abstracts, as well as no fewer than 884 carefully selected relevant sources, have contributed to the success and visibility of these publications.

We would like to thank all 39 authors who contributed to the nine highly up-to-date articles included in this volume, in the form of systematic and narrative reviews, retrospective studies and a study protocol (Table 1).

Patents: Paditz, E. Chronobiologische Säuglingsnahrung und Verfahren zu deren Herstellung. WIPO/PCT/WO2023134856A1. Available online: https://worldwide.espacenet.com/patent/search/family/080123321/publication/WO2023134856A1?q=Paditz (accessed on 20 July 2023).

## Figures and Tables

**Table 1:** Key content of the Special Issue ‘Current Advances in Pediatric Sleep Medicine’, Volume 1, along with data detailing its reception (views and citations, as of 25 July 2026).

Number	Author	Number of Authors	Countries	Type	Tables, Figures, Graphical Abstract	References	Views	Citations	Content
1	Paditz E	1	Germany	Systematic review	6	238	12,499	23	Melatonin, pineal gland, infancy
2	Romero-Peralta S et al.	10	Spain	Systematic review		71	4059	10	OSA, gender differences
3	Müller-Hagedorn S et al.	3	Germany, France	Narrative review	19	177	6920	5	OSA, craniofacial anomalies, airway patency
4	Stuck BA, Schneider B	2	Germany	Narrative review	2	38	368	2	OSA, watchful waiting, partial tonsillectomy
5	Hübler A	1	Germany	Narrative review	4	138	4213	2	Sensory needs, neonatology
6	Schlarb A et al.	6	Germany	Retrospective study, N = 499	9	32	3303	1	Insomnia, nightmares
7	Verbeke J et al.	7	Belgium	Study protocol	1	58	8496	0	Orofacial myofunctional therapy, OSA
8	Upreti K, Frass M	2	India, Austria	Systematic review	8	66	2719	0	Homeopathy, evidence-based medicine
9	Blokland R et al.	7	Australia	Retrospective study, N = 19	7	66	2719	0	Drug-induced sleep endoscopy, residual pediatric OSA
		**39**	**7**		**56**	**884**	**45,296**	**43**	
